# Molecular determinants of anti-EGFR sensitivity and resistance in metastatic colorectal cancer

**DOI:** 10.1038/sj.bjc.6606008

**Published:** 2010-11-23

**Authors:** F Di Fiore, R Sesboüé, P Michel, J C Sabourin, T Frebourg

**Affiliations:** 1 Inserm U614, Faculty of Medicine, Institute for Biomedical Research, 22 Boulevard Gambetta, 76183 Rouen, France; 2 Digestive Oncology Unit, Department of Hepato-Gastroenterology, Rouen University Hospital, 1 rue de Germont, 76031 Rouen Cedex, France; 3 Department of Pathology, Rouen University Hospital, 1 rue de Germont, 76031 Rouen Cedex, France

**Keywords:** colorectal cancer, EGFR, monoclonal antibodies, predictive marker, *KRAS*

## Abstract

Since 2004, the clinical impact of monoclonal antibodies (mAbs) targeting the epidermal growth factor receptor (EGFR) on patients with metastatic colorectal cancer (MCRC) has been clearly established. The combination of these biological agents with conventional chemotherapy has led to a significant improvement in response rate, progression-free survival and overall survival in first-line as well as in second- or third-line treatment of MCRC. However, the high variability of response and outcome in MCRC patients treated with these anti-EGFR mAbs has highlighted the need of identifying clinical and/or molecular predictive markers to ensure appropriate use of targeted therapies. The presence of somatic *KRAS* mutations has been clearly identified as a predictive marker of resistance to anti-EGFR in MCRC, and the use of anti-EGFR mAbs is now restricted to patients with no detectable *KRAS* mutation. Several studies have indicated that amplification of *EGFR*, overexpression of the EGFR ligands and inactivation of the anti-oncogene *TP53* are associated with sensitivity to anti-EGFR mAbs, whereas mutations of *BRAF* and *PIK3CA* and loss of PTEN expression are associated with resistance. Besides these somatic variations, germline polymorphisms such as those affecting genes involved in the EGFR pathway or within the immunoglobulin receptors may also modulate response to anti-EGFR mAbs. Until now, all these markers are not completely validated and only *KRAS* genotyping is mandatory in routine practice for use of the anti-EGFR mAbs in MCRC.

Colorectal cancer (CRC) is the third most common cancer worldwide. Synchronous metastases are estimated to occur in 25% of patients, and ∼40–50% of patients with newly diagnosed CRC develop secondary metastases (Meyerhardt and Mayer, 2005). Since 2004, numerous studies have demonstrated the efficiency of monoclonal antibodies (mAbs) targeting the epidermal growth factor receptor (EGFR) ectodomain, such as cetuximab and panitumumab, in patients with metastatic colorectal cancer (MCRC). Cetuximab corresponds to a chimeric mouse human IgG1 mAb, and panitumumab is a fully human IgG2 mAb.

Indeed, several randomised trials conducted in chemorefractory as well as in chemonaive MCRC patients have reported significant results for cetuximab and panitumumab monotherapy or chemotherapy-based (CT) regimens (Cunningham *et al*, 2004; Jonker *et al*, 2007; Van Cutsem *et al*, 2007, 2009a; Bokemeyer *et al*, 2009). In a randomised phase II trial including irinotecan-refractory MCRC patients, Cunningham *et al* (2004) reported that cetuximab plus irinotecan significantly improved the response rate and progression-free survival (PFS) when compared with cetuximab alone (22.9 *vs* 10.8% and 4.1 *vs* 1.5 months, respectively). Recently, a phase III randomised trial conducted by Van Cutsem *et al* (2009a, 2009b) showed that in chemonaive MCRC patients, the addition of anti-EGFR to irinotecan-based CT lead to an 8.2% increase in the objective response (46.8 *vs* 38.4%), a 0.9-month increase in the PFS (8.9 *vs* 8 months) and a 1.3-month increase in the overall survival (OS) (19.9 *vs* 18.6 months). Although the response to anti-EGFR mAbs observed in some patients has confirmed that EGFR activation is oncogenic, as predicted by cellular and animal models, the molecular mechanisms underlying EGFR activation in colorectal cancer remain obscure and are probably heterogeneous. This situation contrasts with in lung adenocarcinoma in which the key mechanism of EGFR activation underlying sensitivity to EGFR inhibitors corresponds to activating mutations within the EGFR tyrosine kinase domain. Although the use of anti-EGFR mAbs was initially restricted to MCRC patients with a detectable expression of EGFR by immunochemistry (IHC), the lack of IHC predictive value and the heterogeneous clinical response have highlighted the need to identify reliable markers predictive of response to anti-EGFR mAbs (Chung *et al*, 2005).

Two types of molecular predictive markers have been investigated. The majority of studies published so far have analysed somatic alterations affecting effectors of EGFR pathways, such as the receptor–ligand complex, the RAS–RAF–mitogen-activated protein kinase (MAPK) and phosphatidylinositol 3 kinase (PI3K)–Akt–PTEN transduction cascades and p53 (Moroni *et al*, 2005; Lièvre *et al*, 2006, 2008; Benvenuti *et al*, 2007; Di Fiore *et al*, 2007; Frattini *et al*, 2007; Khambata-Ford *et al*, 2007; Amado *et al*, 2008; De Roock *et al*, 2008; Di Nicolantonio *et al*, 2008; Oden-Gangloff *et al*, 2009; Van Cutsem *et al*, 2009a). Studies have indicated that amplification of EGFR and overexpression of the EGFR ligands are associated to sensitivity to anti-EGFR mAbs, whereas mutations of BRAF, of PIK3CA and loss of PTEN expression are associated with resistance (Figure 1, bottom panel). The second type of predictive markers investigated are germline polymorphisms of genes involved in the EGFR pathway (Khambata-Ford *et al*, 2007; Graziano *et al*, 2008; Garm Spindler *et al*, 2009) or within the immunoglobulin receptors, considering the potential role of antibody-dependent cellular cytotoxicity (ADCC) in the action of anti-EGFR mAbs (Zhang *et al*, 2007; Bibeau *et al*, 2009). At the present time, the only molecular marker predictive of the response to anti-EGFR mAbs, which has been unambiguously validated in MCRC by numerous studies, is the somatic mutation of *KRAS* as a marker of resistance to anti-EGFR (Lièvre *et al*, 2006, 2008; Di Fiore *et al*, 2007; Frattini *et al*, 2007; Amado *et al*, 2008; De Roock *et al*, 2008; Van Cutsem *et al*, 2009a). This has led to systematic *KRAS* genotyping in MCRC patients and to the restriction of anti-EGFR mAbs to patients with no detectable *KRAS* mutation. Nevertheless, *KRAS* mutations are obviously not the only determinants of the clinical response to anti-EGFR.

## Overview of the EGFR pathway

**Figure 1:**
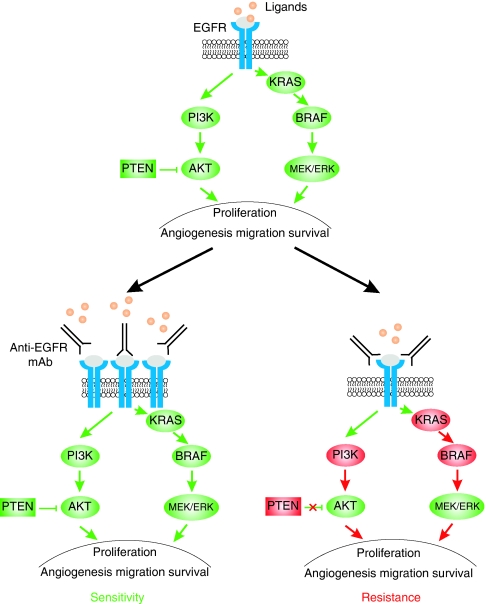
An overview of the EGFR pathway and its main downstream effectors (top). Expected outcomes of anti-EGFR (mAb) therapy (bottom): sensitivity (tumour response) when EGFR is activated (gain copy number, ligand overexpression, other unknown mechanisms) and downstream effectors are wild type (left); resistance (tumour development and metastasis) when downstream effectors such as KRAS, BRAF or PI3K are activated or PTEN is inactivated (right).

The receptors of EGF are composed of homodimers or heterodimers of four related glycoproteins: HER1 (or EGFR), HER2 (or Erbb2), HER3 and HER4 (Figure 1, top panel). These receptors are composed of an extracellular ligand-binding domain, a transmembrane segment and an intracellular protein tyrosine kinase domain. In a normal cell, activation of EGFR is induced by the binding of the ligands to the ectodomain (Ciardiello and Tortora, 2001, 2008). Approximately ten ligands can activate the EGFR pathway. The ligands for HER1/EGFR are EGF, TGF-*α*, HB-EGF, amphiregulin, epiregulin and VGF; heregulins are the ligands of Her-3; NRG2, NRG3, heregulins and *β*-cellulin are the ligands of ErbB-4, and Her-2 is an orphan receptor. Binding of the ligands will induce EGFR homodimerisation (EGFR-EGFR) or heterodimerisation (EGFR-HER2; EGFR-HER3 or EGFR-HER4) and then ATP-dependent phosphorylation of tyrosine residues located within the intracellular domain. The phosphorylated EGFR will then lead to the activation of the RAS–RAF–MAPK kinase and the PI3K–Akt transduction cascades. The RAS–RAF–MAPK kinase is the major downstream signalling route of the EGFR pathway and it controls cell-cycle progression, differentiation, survival and in particular, the G1/S-phase transition (Ciardiello and Tortora, 2008). The RAS proteins are members of a large superfamily of guanine guanosine-5′-triphosphate (GTP) and guanine guanosine-5′-diphosphate (GDP)-binding proteins. In normal cells, EGFR activates RAS by stimulating its binding to GTP. RAF will then be activated and will phosphorylate the MAP2K1 and MAP2K2 kinases. The p110 subunit of PI3K encoded by the *PIK3CA* oncogene is activated by RAS proteins. The PI3K–Akt pathway, which is negatively regulated by the PTEN protein, activates antiapoptotic and survival signals (Figure 1, top panel).

In cancerous cells, EGFR pathway activation results in cell proliferation, inhibition of apoptosis, activation of invasion, metastasis and tumour neovascularisation (Ciardiello and Tortora, 2001, 2008; Cohen *et al*, 2005). The main mechanism of EGFR activation, so far characterised in CRC, corresponds to the somatic mutation of the *KRAS* proto-oncogene. Somatic mutations of *KRAS* are detected in ∼30–40% of CRCs (Andreyev *et al*, 2001). These activating mutations are missense mutations that introduce amino-acid substitutions mainly at positions 12, 13 and 61. Activating mutations of other EGFR pathway effectors have also been described. In CRC, mutations of *BRAF* and *PI3K* genes are detected in ∼5–10% and 6–13% of the tumours, respectively (Moroni *et al*, 2005; Lièvre *et al*, 2006; Di Nicolantonio *et al*, 2008; Laurent-Puig *et al*, 2009; Prenen *et al*, 2009; Sartore-Bianchi *et al*, 2009). Somatic mutations of *KRAS* and *BRAF* are mutually exclusive.

## *KRAS* mutation: a validated predictive marker of resistance to anti-EGFR

Since 2004, the predictive value of somatic *KRAS* mutation, in terms of resistance to anti-EGFR mAbs, has been established by numerous studies. These studies (see Table 1), mainly focusing on mutations of codons 12 and 13, and more recently on codon 61, have been based on molecular analyses of tumour-extracted DNAs from patients included in retrospective studies, as well as in prospective randomised trials (Lièvre *et al*, 2006, 2008; Benvenuti *et al*, 2007; Di Fiore *et al*, 2007; Frattini *et al*, 2007; Amado *et al*, 2008; De Roock *et al*, 2008; Bokemeyer *et al*, 2009; Loupakis *et al*, 2009a; Van Cutsem *et al*, 2009a). The first line of evidence that *KRAS* mutation was a strong predictor of resistance to anti-EGFR mAbs was the observation that in chemorefractory patients treated with cetuximab-based CT, <2% of patients with detectable *KRAS* mutation exhibited an objective response, whereas ∼40% of patients with no detectable *KRAS* mutation showed a clinical response (Lièvre *et al*, 2006, 2008; Benvenuti *et al*, 2007; Di Fiore *et al*, 2007, 2008a, 2008b; Frattini *et al*, 2007; De Roock *et al*, 2008; Loupakis *et al*, 2009a). The impact of *KRAS* status in response to anti-EGFR mAbs was also documented by trials in chemorefractory patients comparing cetuximab or panitumumab monotherapy *vs* best supportive care, with an increase in the response rate in patients with no *KRAS* mutation from 8 to 12.8% and from 10 to 17%, respectively (Karapetis *et al*, 2008).

In chemonaive patients with no detectable *KRAS* mutation, the addition of anti-EGFR antibodies to CT increased the objective response from approximately 46 to 60% when compared with CT alone (Douillard *et al*, 2008, 2009; Bokemeyer *et al*, 2009; Van cutsem *et al*, 2009a). Except in the recent COIN randomised trial (Maughan *et al*, 2009), the addition of anti-EGFR mAbs to CT has also been shown to lead to a significant increase in the median PFS when compared with irinotecan- or oxaliplatin-based CT alone in chemonaive as well as in chemorefractory patients. In contrast, in mutant *KRAS* patients, no clinical benefit was observed from the combination of anti-EGFR plus CT. The effect of *KRAS* status on OS in MCRC patients treated in first line remains debatable, with contradictory results that are mainly explained by the non-standardisation of second- and third-line CT as well as several confounding factors such as treatment crossover and different monitoring methods used in these such randomised trials (Douillard *et al*, 2009; Van Cutsem *et al*, 2009a, 2009b).

## Other somatic predictive markers of response to anti-EGFR

### PIK3CA and PTEN alterations

The demonstration that in MCRC patients *KRAS* mutations confer resistance to anti-EGFR has validated the hypothesis that targeting EGFR will be inefficient if activation of the EGFR transduction cascade results from somatic alterations affecting downstream effectors (Figure 1, bottom panel). This led to an investigation of the predictive value of somatic alterations affecting other effectors. In colorectal cancer, activation of the PIK3CA pathway can result either from activating *PIK3CA* mutations or from inactivation of the PTEN protein. Since 2005, the impact of *PIK3CA* mutations on MCRC patients treated with anti-EGFR has been investigated by several studies that have reported a *PIK3CA* mutation frequency ranging from 6 to 13% (Moroni *et al*, 2005; Lièvre *et al*, 2006; Perrone *et al*, 2009; Prenen *et al*, 2009; Sartore-Bianchi *et al*, 2009). In the studies of Moroni *et al* (2005), Lièvre *et al* (2006) and Perrone *et al* (2009), none of the responders to anti-EGFR therapy had a *PIK3CA* mutation, but this association was not statistically significant, given the limited number of patients. In contrast, a significant association between the presence of *PIK3CA* mutation and response to anti-EGFR has been recently reported by Sartore-Bianchi *et al* (2009) in a study including 110 patients treated with anti-EGFR mAbs. A total of 32 (29%) patients had a *KRAS* mutation and 15 (13%) had a *PIK3CA* mutation. When compared with the unselected population, the response rate to anti-EGFR increased from 20 to 26% in patients with no *KRAS* mutation, to 23% in patients with no *PIK3CA* mutation and to 31% in patients with no *KRAS*/*PIK3CA* mutations (Sartore-Bianchi *et al*, 2009). Nevertheless, these results were not confirmed by the recent study of Prenen *et al* (2009), in which *PIK3CA* mutations were identified in 5 of 39 (13%) responders *vs* 18 of 160 (11%) nonresponders (*P*=0.781). These contradictory results highlight the fact that the association between *PIK3CA* mutations and resistance to anti-EGFR is not yet validated.

*PTEN* exerts an effect as a tumour suppressor by dephosphorylating the plasma membrane lipid second messenger PIP-3 generated by the action of PI3KCA. The loss of PTEN function induces an increase in PIP-3 concentration and PIK3CA pathway activation. IHC and FISH analyses are the two main methods that have been used to evaluate the PTEN status in studies focused on anti-EGFR resistance. However, the molecular mechanisms leading to PTEN inactivation are heterogeneous and include genomic deletions, inactivating mutations and promoter hypermethylation, indicating that PTEN integrity cannot be explored by a simple method. In a series of 27 MCRC patients, Frattini *et al* (2007) reported that PTEN alteration detected by IHC in primary tumours was significantly associated with cetuximab resistance. More recently, Loupakis *et al* (2009b) have analysed by IHC the impact of PTEN cytoplasmic staining intensity in 85 primary tumours and 55 related metastases from patients treated with cetuximab plus irinotecan. First, using a specific scoring system for PTEN expression, these authors found that IHC staining was concordant in only 27 out of 45 (60%) pairs of assessable primary and related metastases. Second, the results of the PTEN IHC performed on the 85 primary tumours did not correlate with the response and PFS, whereas PTEN IHC analysis performed on the 55 metastases was significantly associated with response rate and PFS. When the *KRAS* mutational status in the primary tumours and the PTEN expression in metastasis were combined, the subgroup of patients with no detectable *KRAS* mutation and positive to PTEN had a significantly higher response and longer PFS than patients with *KRAS* mutation and negative to PTEN. In patients with no *KRAS* mutation, Laurent-Puig *et al* (2009) found that the absence of PTEN expression, detected in 19.9% of patients using another IHC scoring system, was associated with shorter OS in univariate and multivariate analyses. In a series of 72 MCRC patients treated with anti-EGFR mAbs plus CT, Razis *et al* (2008) evaluated the correlation of *PTEN* gene copy number and sensitivity to anti-EGFR. The absence of *PTEN* gene dosage alteration was significantly associated with response rate, whereas *PTEN* gene deletion was correlated with worse PFS. No significant association was found using PTEN IHC expression. All these results clearly indicate that no obvious association has yet been established between PTEN alterations and anti-EGFR therapy in MCRC patients. It should also be noted that commercially available anti-PTEN antibodies do not always provide reproducible results in IHC. Further studies are needed to determine the most efficient and reproducible methods for *PI3KCA* and *PTEN* analysis in order to clearly evaluate the predictive value of each marker separately or in combination with *KRAS* status.

### *BRAF* mutation

Two studies support that V600E mutation, resulting in strong activation of the BRAF protein downstream to KRAS, was associated with shorter PFS and OS in MCRC chemorefractory patients treated with anti-EGFR mAbs (Di Nicolantonio *et al*, 2008; Laurent-Puig *et al*, 2009). Nevertheless, it seems that whatever the treatment, this mutation in MCRC is mainly associated with poor prognosis, which may interfere with its predictive value regardless of anti-EGFR mAbs (Tol *et al*, 2010; Van Cutsem *et al*, 2010).

### *TP53* mutations

In a normal cell, the p53 protein exerts an effect not only as a guardian of the genome, which is activated when DNA damage occurs, but also as a policeman of oncogenes, which become active when oncogenes are inappropriately activated, thus inducing apoptosis and/or senescence (Efeyan and Serrano, 2007; Halazonetis *et al*, 2008). Alteration of the p53 pathway has been reported to be systematically observed in non-small cell lung cancer with activating *EGFR* mutations, suggesting that p53 inactivation is required to allow expansion of a cell with EGFR pathway activation (Mounawar *et al*, 2007). On the other hand, it has been shown that PI3K signalling activates p53-mediated growth suppression, suggesting that p53 exerts an effect as a brake for the activated PI3K transduction cascade (Kim *et al*, 2007). Taken together, these data led us to formulate the hypothesis that activation of the EGFR pathway should be oncogenic, and therefore anti-EGFR antibodies should only be efficient in tumours, only if p53 is inactivated. We have recently analysed the impact of *TP53* mutations on 64 MCRC patients treated with cetuximab plus CT (Oden-Gangloff *et al*, 2009). In this series, *TP53* mutations were found in 41 of 64 patients and were significantly associated with controlled disease (*P*=0.037) and higher PFS (20 *vs* 12 weeks, *P*=0.004). In the subgroup of patients without *KRAS* detectable mutation, we have also found that controlled disease and PFS were significantly improved in *TP53* mutated patients when compared with *TP53* nonmutated patients. These results might be explained not only by the fact that EGFR activation is oncogenic only if TP53 is inactivated, but also by the fact that inactivation of TP53 could be one of the mechanisms leading to EGFR activation. Cellular models and studies on larger MCRC series are necessary to clarify the relationship between TP53 status and sensitivity to anti-EGFR.

### *EGFR* gene copy number and expression of its ligands

#### *EGFR* gene copy number

A common mechanism of EGFR activation is the increase in the *EGFR* copy number corresponding to the gain of copies or amplification of the chromosome 7 region on which *EGFR* is located. A copy number increase of *EGFR* resulting from chromosome 7 polysomy probably does not have the same biological significance than amplification of the *EGFR* locus. In contrast to breast cancer in which tumour cells exhibit *HER2* amplification in homogeneous regions with a relationship between expression and gene amplification, colorectal cancer is characterised by a heterogeneous *EGFR* gene copy number pattern and, furthermore, no correlation has been established between the *EGFR* copy number and the EGFR IHC staining. Based on the results obtained in patients with metastatic breast cancer treated with trastuzumab, in which *ERBB2* amplification correlates with sensitivity to mAb therapy, Moroni *et al* (2005) investigated the impact of the *EGFR* gene copy number, assessed by FISH, on the anti-EGFR mAb response in a series of 30 MCRC patients. This study reported a positive significant association between an increased *EGFR* gene copy number and the response rate to cetuximab or panitumumab. Since 2005, many reports have investigated the relationship between EGFR copy number and response to mAb therapy in MCRC, but only five of them restricted the analysis to the subgroup of wild-type *KRAS* patients (Table 2). Although an increase in EGFR copy number is significantly associated with mAb therapy response, the results are less obvious in terms of PFS or OS. Cappuzzo *et al* (2008), in a series of 85 chemorefractory patients treated with cetuximab, determined that the best cutoff significantly associated with the response rate and PFS was 2.92 *EGFR* gene copy number per cell. Interestingly, these authors further analysed their population according to the *EGFR* copy number cutoff previously reported by Sartore-Bianchi *et al* (2009) and observed a significant association for response rate but not for PFS. It can be concluded from the different studies published so far that only a high *EGFR* gene copy number, resulting from gene amplification and observed in a small fraction of patients, is probably predictive of anti-EGFR sensitivity in MCRC patients.

#### Expression of EGFR ligands

Few studies have investigated the predictive role of EGFR ligand expression on anti-EGFR response in MCRC. A transcriptome analysis of 164 CRCs, using a 640 probe set, showed that the EGFR ligands, epiregulin (*EREG*) and amphiregulin (*AREG*), were highly expressed in 25% of the samples (Khambata-Ford *et al*, 2007). In this study, analysis of metastatic frozen samples from a population of 80 MCRC patients treated with cetuximab monotherapy showed that patients with disease control expressed the EGFR ligands at a higher level than nonresponders. Moreover, patients with tumours exhibiting higher *EREG* or *AREG* expression had significantly longer PFS, suggesting that these tumours were EGFR dependent and were therefore particularly sensitive to the inhibition of the ligand–receptor interaction by anti-EGFR mAb (Khambata-Ford *et al*, 2007). In a recent study performed on paraffin-embedded samples in refractory MCRC patients treated with cetuximab and irinotecan, the *KRAS* status and the *EREG* and *AREG* mRNA expression were first determined on 220 primary samples and the results were then validated on a series of 67 patients (Jacobs *et al*, 2009). The mRNA levels of *EREG* and *AREG* significantly correlated with the absence of detectable *KRAS* mutation. When ligand expression level was combined with the *KRAS* status, the median OS was 65 weeks in patients with no detectable *KRAS* mutation and a high EREG expression *vs* 31 weeks (*P*=0.01) in patients without detectable *KRAS* mutation and low EREG expression (Jacobs *et al*, 2009).

## Germline polymorphisms associated with clinical response to anti-EGFR mAbs

Three types of germline polymorphisms suspected of modulating the response to anti-EGFR have been investigated. The first corresponds to a CA repeat present within the *EGFR* intron 1, which has been shown to modulate EGFR transcription efficiency, the longer (L) repeats being associated with a reduced transcription when compared with the shorter (S) ones. The second, located within the *EGF* gene, corresponds to a SNP (G>A, rs4444903). In 110 chemorefractory MCRC patients treated with cetuximab–irinotecan, Graziano *et al* (2008) showed that *EGFR* intron-1 variant (S/S *vs* L/L and L/S) and *EGF61* variant (G/G *vs* A/A and A/G) were significantly associated with a better OS. Similar results were obtained on *EGF61* genotypes in a study on chemorefractory MCRC patients treated with cetuximab plus irinotecan CT (Garm Spindler *et al*, 2009). In patients with no detectable *KRAS* mutation, homozygous phenotypes (A/A and G/G) were associated with a lower progression rate (19 *vs* 60%) than EGF61 A/G patients (*P*=0.006) and a significant increase in OS (17.1 *vs* 5.9 months). The third type of germline polymorphism investigated, in the context of anti-EGFR therapies in MCRC, corresponds to polymorphic amino-acid substitutions within the receptors of the Fc fragment of the immunoglobulins (Igs). The H131R and V158F variations within the Fc*γ* RIIa and Fc*γ* RIIIa have been shown to modify the affinity of the Ig receptors and are therefore predicted to modulate the ADCC in patients receiving anti-EGFR IgG1 mAb. To date, only four studies have found significant association between these polymorphisms and the clinical outcome. In a first study performed in a series of 39 chemorefractory patients treated with cetuximab monotherapy, Zhang *et al* (2007) found that the presence of the *FCGR2A* 131 H allele and of the *FCGR3A* 158 F allele were significantly associated with better PFS; however, these results could not be replicated on a larger series including 130 subjects (Lurje *et al*, 2008). In 64 chemorefractory MCRC patients treated with cetuximab-based CT, Bibeau *et al* (2009) found that the *FCGR2A* 131 H/H and *FCGR3A* 158 V/V genotypes, predicted to result in a higher affinity of the Ig to the receptor, were significantly associated with better PFS. In this study, analysis of these germline polymorphisms in the subgroup of patients with no detectable *KRAS* mutation showed that the median PFS was 9.6 months in the 131 H/H or 158 V/V patients *vs* 4.6 months in the subgroup of patients harbouring the other genotypes (*P*=0.015). In contrast, a more recent study (Pander *et al*, 2010), performed on KRAS wild-type patients receiving chemotherapy plus cetuximab, showed that subjects homozygous for the FCGR3A 158 F allele have a better PFS than those bearing other allelic combinations. Several factors may account for these discrepancies; although ethnic origin is unlikely to be involved, previous treatment regimens and association of irinotecan with cetuximab could modify patients’ response. Further studies involving testing integrity of the EGFR pathway are clearly needed to clarify this point.

## Mutational status in primary colorectal adenocarcinomas and related metastases

One of the main subjects of debate is the relevance of *KRAS* genotyping on primary tumours, whereas anti-EGFR mAbs are used to treat a metastatic disease. Indeed, some publications have indicated that concordance of mutational status of KRAS and other EGFR downstream effectors between primary and metastases is not absolute. In a series of 48 CRC patients, an overall concordance of 92% was observed between primary tumours and metastases for the presence of mutations within *KRAS* exon 2 and *BRAF* exon 5 (Artale *et al*, 2008); however, in the specific subgroup of patients with KRAS mutation, 23% of discordance was observed between the two tumour sites. In a study including 38 MCRCs, Molinari *et al* (2009) reported a concordance for *KRAS* and *BRAF* mutational status of 92 and 100%, respectively. More recently, the analysis of *KRAS*, *BRAF* and *PIK3CA* in tumour invasion fronts, lymph nodes and distant metastases revealed a discordance between primary tumours and lymph node metastases of 31, 4 and 13%, respectively (Baldus *et al*, 2010). A discordance between primary tumours and distant metastases was observed for *KRAS* in 10% of the cases and for PIK3CA in 5% of the cases (Baldus *et al*, 2010). In a large series of >800 samples tested for *KRAS*, using a highly sensitive SNaPshot-based method (Di Fiore *et al*, 2007), we observed a discordant *KRAS* mutational status between tumours and metastases in 10% of the paired samples (Lamy *et al*, 2010). These studies clearly indicate that given the genetic heterogeneity and genetic evolution of CRC under treatment, the most appropriate tissue on which molecular profiling should be performed remains to be determined.

## Conclusion and perspectives

In conclusion, the numerous studies published since 2006 on the markers of sensitivity and resistance to anti-EGFR mAbs in MCRC have clearly validated somatic *KRAS* mutations as markers of resistance to anti-EGFR, resulting in restricted use of these targeted therapies to patients with no detectable *KRAS* mutation. However, in these patients, the heterogeneity of response strongly suggests that several genetic variations, corresponding either to somatic alterations present only within the tumours or to germline polymorphisms, can modulate sensitivity or resistance to anti-EGFR mAbs. One of the challenges is now to identify these other genetic predictive markers, using either candidate gene approaches, global analysis based on pangenomic/transcriptomic/proteomic portraits or cellular or animal models. Some technical and conceptual questions concerning *KRAS* genotyping remain unsolved. One important question under debate is to define in MCRC patients, the most appropriate tissue on which DNA analysis should be performed in the context of targeted therapies. Indeed, most studies published so far are based on the analysis of genetic material extracted from primary tumours, whereas targeted therapies are mainly directed at metastatic disease treatment. As highlighted by several recent studies, this approach may have limitations: (1) the primary tumour may not always be available; (2) as indicated above, several genetic changes may occur between the primary tumour and the corresponding metastases; and (3) genetic analyses performed on paraffin-embedded tissues may generate artefacts because of fragmentation and chemical modification of DNA. Therefore, we think, as previously shown, that genetic analysis based on highly sensitive detection from blood of circulating mutant DNA might be of clinical interest (Di Fiore *et al*, 2008a; Yen *et al*, 2009).

As the new markers will have to be validated by independent studies, the challenge for the clinician, before treating patients, will be the progressive integration of these markers in the routine and the development of a decisional algorithm, which should probably be stratified first by *KRAS* genotyping. In patients with *KRAS* mutations, the choice of the most appropriate treatment is problematic. Although the use of anti-EGFR mAbs is not recommended in these patients, it should be underlined that disease stability can be observed in up to 50% of chemorefractory patients treated with anti-EGFR alone in association with CT (Di Fiore *et al*, 2008b). In these patients, targeted therapies will probably be based, in the future, on molecules able to inhibit effectors of the EGFR transduction cascade and located downstream from the KRAS protein.

## Figures and Tables

**Table 1:** Response rate (OR) and PFS in MCRC patients treated with anti-EGFR mAb-based CT

**Regimen(s)**	**Study**	***n***	**Response KRAS WT (%)**	***P-*value**	**PFS KRAS WT (months)**	***P-*value**
*Chemonaive patients: 1st line*						
Cmab+FOLFOX *vs* FOLFOX	Bokemeyer *et al* (2009, 2010)	315	57.3 *vs* 34.0	0.0027	8.3 *vs* 7.2	0.0064
Cmab+FOLFIRI *vs* FOLFIRI	Van Cutsem *et al* (2009a, 2009b, 2010)	1063	57.3 *vs* 39.7	<0.0001	9.9 *vs* 8.4	<0.0012
Pmab+FOLFOX *vs* FOLFOX	Douillard *et al* (2009)	1183	55.0 *vs* 48.0	NS	9.6 *vs* 8.0	0.02
						
*Chemorefractory patients: 2nd line*						
Pmab + FOLFIRI *vs* FOLFIRI	Peeters *et al* (2009)	1186	35 *vs* 10	<0.01	5.9 *vs* 3.9	0.004
						
*Chemorefractory patients: ⩾2nd line*						
Cmab±CT	Lièvre *et al* (2006)	30	40.7	—	—	—
Cmab±CT	Di Fiore *et al* (2007)	59	27.9	—	5.5	—
Cmab±CT	Lièvre *et al* (2008)	114	43.5	—	7.4	—
Cmab±CT	De Roock *et al* (2008)	80	45.8	—	7.9	—
Cmab/Pmab±CT	Benvenuti *et al* (2007)	48	31.2	—	—	—
						
*Chemorefractory patients:* >*3rd line*						
Cmab mono *vs* BSC	Karapetis *et al* (2008)	394	12.8 *vs* 0	—	3.7 *vs* 1.9	<0.001
Pmab mono *vs* BSC	Amado *et al* (2008)	427	17 *vs* 0	—	2.9 *vs* 1.7	<0.0001

Abbreviations: BSC=best supportive care; CI=confidence interval; Cmab**=**cetuximab; CT=chemotherapy; EGFR=epidermal growth factor receptor; mAb=monoclonal antibody; MCRC=metastatic colorectal cancer; NS=not significant; OS=overall survival; PFS=progression-free survival; Pmab=panitumumab; WT=wild type.

**Table 2:** Influence of *EGFR* status (gene copy number) on subject response in *KRAS* wild-type MCRC patients treated with anti-EGFR mAb-based CT

			**PFS (months)**			**OS (months)**			**Responders**	**Nonresponders**	
**Study**	***N***	**EGFR cutoff**	**No**	**95% CI**	***P-*value**	**No**	**95% CI**	***P-*value**	**CR+PR**	**SD+PD**	***P-*value**
Moroni *et al* (2005)	20	⩾3^a^	—			—			6	1	0.001
		<3	—			—			1	12	
Personeni *et al* (2008)	58	⩾2.83^a^	5.5		0.25	10		0.037	17	10	0.007
		<2.83	4			8.3			8	23	
Laurent-Puig *et al* (2009)	116	⩾2^a^	8.5	7–16	0.28	20	13–NA	0.018	12	5	0.015
		<2	7	5–8.5		14	11–17		29	50	
Scartozzi *et al* (2009)	44	⩾2.6^a^	7.7		0.04	16		0.2	9	6	0.001
		<2.6	2.9			9.5			2	21	
		⩾2.12^b^	6.4		0.02	10.6		0.95	10	18	0.04
		<2.12	3.1			10.3			1	15	
Tol *et al* (2010)	155	⩾3^a^	9.5	6.4–12.3	0.19	21.9	13.4–27.1	0.65	—	—	
		<3	10.4	8.6–12.2		22	19.2–25.4		—	—	

Abbreviations: CI=confidence interval; CISH=chromogenic *in situ* hybridisation; CR=complete response; CT=chemotherapy; EGFR=epidermal growth factor receptor; FISH=fluorescence *in situ* hybridisation; mAb=monoclonal antibody; MCRC=metastatic colorectal cancer; NA=not assessable; OS=overall survival; PFS=progression-free survival; PR=partial response; PD=progressive disease; SD=stable disease.

a FISH.

b CISH.
